# Eye-Tracked Visual Attention to Anthropomorphic Appearance and Empathic Responses in AI Medical Conversational Agents: Dissociating Trust Gains from Attentional Synergy

**DOI:** 10.3390/jemr19020038

**Published:** 2026-04-09

**Authors:** Wumin Ouyang, Hemin Du, Yong Han, Zihuan Wang, Yuyu He

**Affiliations:** 1 School of Design and Innovation, Shenzhen Technology University, Shenzhen 518118, China; u23092110115@cityu.edu.mo (W.O.);; 2 Faculty of Innovation and Design, City University of Macau, Macau 999078, China; 3 College of Engineering and Design, Hunan Normal University, Changsha 410012, China; 4 Waikato Management School, University of Waikato, Hamilton 3240, New Zealand

**Keywords:** AI medical conversational agents, appearance anthropomorphism, empathic response, visual attention, trust

## Abstract

Understanding how users perceive and attend to the anthropomorphic appearance and empathic responses of artificial intelligence medical conversational agents (AIMCAs) can help reveal the key judgment cues underlying trust formation and use decisions, while also informing interface and dialog design. To this end, this study employs a 3 (appearance anthropomorphism: high, medium, low) × 2 (empathic response: present, absent) within-subject eye-tracking experiment, combined with subjective scales and brief post-task open-ended feedback. During a static prototype viewing task based on hypothetical consultation scenarios, we concurrently recorded trust, behavioral intention, and visual measures for key areas of interest (AOIs; appearance area, conversational content area, and overall interface area). Eye-tracking measures were normalized by AOI coverage proportion to improve cross-AOI comparability. The results show that both anthropomorphic appearance and empathic response significantly increased users’ trust in AIMCAs and their behavioral intention. An interaction between these two types of social cues was also observed, suggesting that when visual embodiment and linguistic style are aligned at the social level, users are more likely to form favorable overall judgments. At the level of visual processing, however, no interaction effect was found, and the eye-tracking measures showed only partial main effects, indicating that subjective synergy does not necessarily correspond to synergistic changes in attentional allocation. Overall, anthropomorphic appearance and empathic response exerted consistent facilitating effects on outcome variables, but displayed different patterns of attentional allocation and information prioritization at the visual level. Accordingly, AIMCA design should emphasize consistency between appearance cues and conversational strategies, optimize users’ initial judgments and interface comprehension, and use intention through verifiable information organization and clear boundary cues.

## 1. Introduction

Conversational agents (CAs) generally refer to a class of intelligent software systems with which users can interact through natural language, such as text or speech, and their advancing capabilities have been driven largely by developments in artificial intelligence, particularly natural language processing [1]. In recent years, CAs have gradually evolved from early script-based interaction tools into digital service gateways capable of supporting information retrieval, process guidance, and task execution, and have been rapidly deployed across a wide range of domains, including e-commerce, customer service, education, government, and healthcare services [1,2]. In the healthcare domain, public-facing AI medical conversational agents (AIMCAs) are increasingly being used for tasks such as health consultation, symptom assessment and triage guidance, preoperative and postoperative follow-up support, health education, and behavior promotion, demonstrating considerable potential for improving service accessibility and interaction efficiency [3,4]. Notably, the introduction of generative artificial intelligence and large language models has enabled conversational agents to produce responses that more closely resemble human expression and, to some extent, to support more natural and information-rich clinician–patient-style communication. This development has, in turn, accelerated the transition of medical conversational agents from question-answering and information retrieval tools to more interactive forms of health service delivery and support [4,5]. In parallel, the evidence base in this area has continued to expand. For example, a rapid review covering the literature published between 2017 and 2023 examined the roles and boundaries of medical chat agents in pathways such as telehealth service provision and administrative support [4]. At the same time, a systematic review focusing on large language model-based health advice and health-question answering has synthesized a substantial body of empirical research, indicating that both research and practical interest in generative medical conversational systems are rising rapidly [6]. Against this background, user-centered empirical research on AIMCAs is of considerable importance, because it can not only provide actionable behavioral evidence for interaction design and system optimization, but also offer an empirical foundation for subsequent system evaluation and iterative design in more realistic contexts of use.

Although the application landscape of AIMCAs continues to broaden, both cross-national surveys and China-specific evidence indicate a substantial gap between public trust and adoption of healthcare systems and medical AI. In a nationally representative survey in the United States, 65.8% of respondents reported low confidence that their healthcare system would use AI responsibly, and 57.7% were not confident that the system could ensure that AI tools would not harm them [7]. In the Chinese context, a nationwide multicenter survey targeting individuals with healthcare needs revealed a similar “willing to try, but insufficient trust” pattern: 61.7% of respondents stated that they would be willing to try AI-involved diagnostic and treatment services, yet only 43.5% reported trusting the diagnostic results [8]. At present, the real-world penetration of AIMCAs in China remains limited [9]. Also, evidence on healthcare AI more broadly converges on the view that trust and adoption are jointly constrained by perceived risk, responsibility attribution, transparency, privacy and security, and perceived competence and reliability [10,11]. Against this backdrop, enhancing the trustworthiness of AIMCAs depends not only on algorithmic performance, but also—critically—on the social cues conveyed through the human–AI interaction layer.

Because AIMCAs are fundamentally dialog-centered and continuously present themselves through both interface design and language, users often interpret them as socially relevant interaction partners and therefore rely on perceivable social cues to infer their trustworthiness and adoptability. One important category of such cues is anthropomorphic appearance, which can shape users’ judgments of the system and, in turn, influence trust formation. In medical triage conversational agents, enhancing anthropomorphic cues has been shown to strengthen social presence and facilitate trust development, thereby improving adoption-related outcomes such as continued use [12]. Consistent with this, eye-tracking evidence has also shown that when conversational agents combine a more anthropomorphic appearance with empathic responses, users report higher levels of social presence, trust, and satisfaction [13]. Another important category of cues is empathic response, whose role is typically reflected in increasing perceived warmth and the sense of being understood, and thereby influencing users’ trust in and acceptance of AIMCAs. Experimental research on medical chat agents has directly shown that, compared with non-empathic responses, the inclusion of empathic expressions increases users’ perceived warmth, which in turn leads to greater trust and stronger use intention. At the same time, when such empathic expressions are perceived as insufficiently authentic, their positive effects are attenuated, suggesting that empathic cues in medical contexts are subject to important authenticity-boundary conditions [14]. Building on this, recent evidence from real patient evaluations, comparisons by independent evaluators, and systematic reviews has provided further convergent support: the linguistic empathy of AI chat agents can be recognized by users and can significantly influence evaluations of response empathy, quality, trust, and overall experience through validation, reassurance, non-judgmental expression, and clearer supportive structures [15,16,17]. Therefore, the theoretical foundation for empathic response in the present study is based, on the one hand, directly on Seitz’s experimental test of the “warmth–trust/use intention–authenticity boundary” mechanism and, on the other hand, incorporates the shared support provided by several recent studies for the broader conclusion that empathy at the linguistic level can be recognized and can affect evaluative outcomes [14].

Overall, prior work converges on the view that anthropomorphic appearance and empathic responding constitute two key social cues that are designable and manipulable, and that they may shape adoption-related outcomes of AIMCAs through mechanisms such as social presence, perceived warmth, and credibility. However, existing studies have often examined anthropomorphism and empathic cues in isolation, and empirical evidence on their joint effects and interaction mechanisms in AIMCA settings remains relatively limited. Moreover, most studies rely primarily on self-report measures, which makes it difficult to uncover users’ attentional allocation to these cues and the underlying information-processing processes. In human–computer interaction research, eye tracking can provide more process-proximal, objective indicators at the level of areas of interest (AOIs). It has been used to test how anthropomorphic cues in chat agents influence visual attention and subjective perceptions [13], and it is also regarded in trust research as a complementary source of evidence for capturing dynamic processing [18]. Accordingly, concurrently manipulating an AIMCA’s anthropomorphic appearance and empathic responding and integrating self-report scales with eye-tracking metrics to examine both main and interaction effects has clear theoretical and practical significance.

From a theoretical perspective, this study conceptualizes anthropomorphic appearance and empathic response as two types of social cues that operate through different channels. Anthropomorphic appearance primarily functions as a highly salient visual social cue. It first influences whether users perceive an AIMCA as an interactive entity with social attributes rather than merely as an information tool. This perception then enters users’ judgments of credibility and acceptability by enhancing perceived anthropomorphism and social presence. Research on medical conversational agents has shown that a more humanlike appearance and more humanlike modes of expression can increase perceived anthropomorphism and social presence, thereby further promoting trust formation and acceptance-related outcomes [12,19]. In this sense, anthropomorphic appearance does not simply alter whether the interface “looks human”; more fundamentally, it influences at an earlier stage whether users treat the system as an entity open to social interpretation.

By contrast, empathic response is primarily a linguistic and relational cue. It does not mainly determine whether users regard an AIMCA as a social entity; rather, it more directly shapes whether users feel understood and supported, and whether the interaction is experienced as warm and relationally meaningful. In research on medical chat agents, empathic expression has generally been found to enhance trust and use intention by increasing perceived warmth. However, when such empathy is experienced as inauthentic, its positive effect is attenuated, indicating that empathic cues are not necessarily more effective when they are stronger, but instead need to remain appropriately aligned with the role characteristics of the AI system and users’ interaction expectations [14]. More broadly, research on AI chatbots suggests that trust formation does not depend solely on system performance, but is jointly shaped by machine characteristics, interactional cues, social cues, and contextual factors [20,21].

Based on this logic, the present study defines anthropomorphic appearance and empathic response in AIMCAs as two designable types of social cues: the former primarily contributes to the activation of perceived anthropomorphism and social presence, whereas the latter primarily contributes to the formation of perceived warmth, the sense of being understood, and relational evaluations. Together, these cues enter users’ integrated judgment of trust in AIMCAs and behavioral intention. Accordingly, this study manipulated appearance anthropomorphism (high/medium/low) and empathic response (present/absent) as two within-subject factors, used trust and behavioral intention as outcome variables, and employed AOI-level visual attention as a process indicator to examine whether these two types of social cues exert distinct effects, whether they produce an enhancing effect at the level of subjective judgment, and whether such effects exhibit the same structure at the level of visual processing. This design helps establish a more direct correspondence between the theoretical chain of “social cues–social presence/relational evaluation–trust and behavior” and the specific experimental manipulations.

Given that this study adopts a controlled experimental paradigm, it was not feasible to track participants’ sustained use behavior in real-world contexts within a short period of time. Therefore, behavioral intention to use was treated as one of the key adoption-related outcome variables. This approach is consistent with the dominant paradigm in research on health behavior and technology adoption [22]. At the same time, existing empirical studies on conversational AI have shown that users’ use intention is positively associated with their subsequent actual use, and that use intention can, to some extent, mediate the effect of trust on actual use [23]. It should be noted, however, that a gap may exist between intention and behavior, and that the stability of intention influences its predictive power for behavior [24]. Therefore, the present study further incorporates process-oriented indicators, such as eye-tracking measures, to complement self-report data and to provide evidence more closely related to underlying processing mechanisms for differences in trust and intention. In the present study, behavioral intention was primarily used to capture users’ early acceptance judgments under controlled experimental conditions, whereas its relationship with actual reliance, continued use, and decision quality will need to be further examined in more realistic interactive settings.

To address the above limitations, the present study focuses on AIMCAs and employs a 3 (anthropomorphic appearance: high/medium/low) × 2 (empathic response: present/absent) eye-tracking experimental design. By combining subjective scales with eye-tracking measures, the study examines the effects of anthropomorphic and empathic cues at both the level of outcome variables and the level of process evidence. Based on the theoretical foundations and prior evidence reviewed above, the following hypotheses and research question were proposed:

**H1a:**  *Higher levels of anthropomorphic appearance will increase users’ perceived trust in AIMCAs.*

**H1b:**  *Higher levels of anthropomorphic appearance will increase users’ behavioral intention toward AIMCAs.*

**H2a:**  *AIMCAs with empathic responses will elicit higher perceived trust than AIMCAs without empathic responses.*

**H2b:**  *AIMCAs with empathic responses will elicit higher behavioral intention than AIMCAs without empathic responses.*

**H3a:**  *Anthropomorphic appearance and empathic response will show an enhancing effect on perceived trust; when a higher level of anthropomorphic appearance and empathic response are presented together, users will report the highest perceived trust.*

**H3b:**  *Anthropomorphic appearance and empathic response will show an enhancing effect on behavioral intention; when a higher level of anthropomorphic appearance and empathic response are presented together, users will report the highest behavioral intention.*

RQ1: At the level of visual processing, how do anthropomorphic appearance and empathic response alter users’ attentional allocation across key AOIs, and how can these changes provide process-level evidence for understanding differences in subjective evaluations?

Through this design, the present study makes three contributions. First, it provides joint evidence on the combined role of “appearance cues × communication cues” in the AIMCA context, thereby responding to the need for research examining the potential interaction between anthropomorphic and empathic cues. Second, it incorporates process-oriented eye-tracking measures to reduce the limitations of relying solely on self-report scales when interpreting attentional allocation and information processing. Finally, it offers actionable design implications for levels of anthropomorphic appearance and strategies for empathic response in AIMCAs, with particular emphasis on supporting interface and dialog optimization at the prototype stage and on informing the development of trust and use judgments that are appropriately aligned with system capabilities.

The remainder of this paper is organized as follows. Section 2 reports the methods, including the stimuli and experimental manipulations, participant information, apparatus, procedure, and the self-report and eye-tracking measures. Section 3 presents the empirical results, reporting self-report outcomes and eye-tracking findings, respectively. Section 4 discusses the results, focusing on the effects of anthropomorphic appearance and empathic responding on users’ perceptions (trust and behavioral intention) as well as on visual behavior. Section 5 summarizes implications, limitations, and directions for future research. Section 6 concludes the paper.

## 2. Methodology

### 2.1. Stimuli

This study adopts a 3 (level of appearance anthropomorphism: high anthropomorphism, medium anthropomorphism, and low anthropomorphism) × 2 (empathic response: present vs. absent) within-subject experimental design. An eye-tracking experiment was conducted to investigate users’ visual behavior. During the experiment, participants’ perceptual data were collected through subjective evaluations.

This study uses static interface prototypes as the experimental stimuli rather than allowing participants to engage in free conversational interaction with the AIMCA. Static prototypes were selected for two reasons. First, static interfaces allow visual presentation to be strictly standardized, which facilitates the stable definition of AOIs in eye-tracking analysis and thus yields more reproducible indicators of gaze allocation and processing. By contrast, dynamic videos or free interaction introduce additional sources of variance, such as changes in interface element positions, differences in interaction paths, and system fluctuations, thereby substantially increasing the difficulty of AOI annotation and data interpretation [25,26]. Second, in research on health conversational agents and chat agents, scenario-based prototype experiments are commonly used to isolate the effects of specific social cues on trust and adoption-related outcomes, and are particularly suitable for achieving highly controlled and interpretable causal inference at the early stage of mechanism testing [12,27]. Therefore, the evidence provided in this study primarily pertains to interface prototype viewing, first-impression formation, and short-term evaluative processes under hypothetical consultation scenarios.

The stimulus materials were constructed into six experimental conditions in accordance with a 3 × 2 design. This manipulation corresponds to the theoretical framework of the present study: appearance anthropomorphism primarily carries visual social cues, whose role was expected to be reflected first in strengthening users’ perceptions of AIMCAs as humanlike and socially present entities; empathic response primarily carries linguistic and relational cues, whose role was expected to be reflected mainly in enhancing users’ perceived warmth and sense of being understood. These two types of cues were further assumed to jointly influence users’ trust formation and usage judgments regarding AIMCAs, and were therefore specified as the two core experimental factors in this study [12,14,19]. First, the manipulation of appearance anthropomorphism was based on the graded approaches commonly adopted in prior research on anthropomorphic cues in chat agents [12,27]. Specifically, the high-anthropomorphism condition used a real human face avatar, the medium-anthropomorphism condition used a cartoon humanlike avatar, and the low-anthropomorphism condition used a nonhuman abstract icon, thereby creating a graded variation from strong to weak social cues while keeping interface layout, color, font size, and information structure constant. In addition, gender presentation was standardized across all conditions by adopting a female representation. Previous research has shown that gendered cues in chat agents can systematically influence users’ judgments of warmth, competence, trust, and intention to use, although such effects are not unidirectional or fixed, but are jointly moderated by interaction context, task characteristics, and user attributes. More specifically, gender congruence between a chat agent and its user can enhance users’ sense of self-consistency with the agent and further improve related behavioral responses [28]. In research on intelligent voice assistants, the overall main effect of gender cues has not always been stable, but interactions with task type and user gender may occur; for example, male users may show a greater tendency to trust male voices on certain dimensions of trust [29]. At the same time, even when gender-ambiguous designs are adopted, users still tend to assign gender spontaneously, and those who perceive the agent as female may show higher intention to use it [30]. In the context of medical chat agents, designs incorporating female doctor cues have also been found to more readily elicit both cognitive trust and affective trust, whereas male doctor cues appear to enhance cognitive trust to a greater extent [31]. On the basis of this evidence, the present study adopts female gender presentation as a uniform background social cue in order to establish a relatively stable prototype setting that is more consistent with the medical communication context, while avoiding potential confounding caused by simultaneous variation in gender cues, degree of appearance anthropomorphism, and empathic response. As a result, the analysis in this study can focus more directly on the effect structure of the two core design cues, namely anthropomorphic appearance and empathic response.

Second, the present study defines empathic response as a form of linguistic presentation that involves recognizing and acknowledging users’ emotional cues, expressing understanding and empathy for their situation, and, on this basis, providing supportive and action-oriented assistance. In the empathic-response condition, the AIMCA systematically incorporated into its key replies: (1) recognition and labeling of emotions (e.g., “It sounds like you may be feeling worried/unwell”), (2) expressions of understanding and reassurance (e.g., “This is quite common, and your concern is understandable”), and (3) assistance that combines supportive language with action-oriented suggestions (e.g., “We can first …; if … occurs, it is advisable to seek medical attention promptly”). In the non-empathic-response condition, the AIMCA retained the same medical information and task steps, but removed expressions of emotional acknowledgment, reassurance, and care, instead delivering questions and recommendations in a more neutral, information-oriented manner. In terms of the rationale for this manipulation, the design of empathic response in the present study is based primarily on Seitz’s experimental findings in the context of medical chat agents [14]. That study directly shows that, compared with non-empathic responses, the inclusion of empathic expressions increased perceived warmth and, in turn, enhanced trust and intention to use. At the same time, stronger forms of experiential empathic expression could weaken these positive effects when they were perceived as insufficiently authentic. Accordingly, the present study does not seek to create highly intense or overly emotional expressions in the empathic condition, but instead adopts a more supportive, help-oriented linguistic style that includes moderate emotional acknowledgment. In addition, other recent studies have shown that validation, reassurance, non-judgmental wording, and clear structure in AI responses can indeed be recognized by users as more empathic, and can significantly shape their evaluations of response quality, trust, and overall experience [15,17]. On this basis, the present study conceptualized empathic response as a designable linguistic social cue and operationalized it through a relatively restrained yet strongly supportive mode of expression.

To reduce stimulus-specific bias arising from a single interface configuration and to obtain a more robust assessment of users’ perceptions and visual behavior, this study designed three similar prototypes for each level of anthropomorphism. All high-fidelity prototypes were created using Adobe Illustrator (v24.0), resulting in a total of 18 stimuli (see Appendix A). This approach is consistent with methodological recommendations on stimulus sampling and on improving the generalizability of findings [32]. The combinations of experimental prototypes are presented in Table 1. In the formal statistical analyses, the three same-level prototypes were treated as parallel stimulus instances within the same experimental condition and, after random presentation, were aggregated for condition-level comparison. This treatment is consistent with the present study’s focus on fixed condition effects and also accords with the pilot findings indicating only minor differences among the three prototypes within the same anthropomorphism level.

To further clarify how the stimulus structure was handled in the formal analyses, each participant viewed all 18 interface prototypes during the experiment, and the presentation order was randomized for each participant to reduce order effects and bias caused by the successive appearance of particular prototypes. In the formal statistical analyses, both subjective evaluations and eye-tracking measures were aggregated at the condition level; that is, the main and interaction effects of the two experimental factors, appearance anthropomorphism and empathic response, were estimated without reporting the three parallel prototypes as separate fixed conditions. This approach is consistent with the central aim of the study, namely, to identify the stable effect structure of designable social cues at the condition level. Multi-stimulus sampling helps reduce the incidental influence of any single interface instance on the results and allows condition effects to be estimated on the basis of a broader stimulus set. At the same time, the way in which stimulus instances are incorporated also affects the scope of inference that can be drawn from the findings [32,33].

Before the formal experiment, we conducted a pilot study between 2 and 5 December 2025, to verify the effectiveness of the manipulations and to rule out potential confounds. A total of 30 participants were recruited, aged 20–29 years (M = 26.63, SD = 2.45). All participants had good Chinese reading proficiency and had used online medical consultation services within the past six months. Each participant viewed the 18 interface prototypes online one by one and provided a brief evaluation after each stimulus.

To test the manipulation of anthropomorphic appearance, we used a 7-point Likert scale commonly adopted in medical conversational agent research to assess perceived anthropomorphism [12] (e.g., “The appearance of this AIMCA makes me feel that it is very human-like,” “The appearance of this AIMCA makes me feel it has ‘human characteristics/human temperament’”). The repeated-measures ANOVA indicates significant differences across the three levels of appearance anthropomorphism [F(2, 58) = 114.843, *p* < 0.001]. Specifically, perceived anthropomorphism differed significantly among the high-anthropomorphism (M = 4.92, SD = 0.91), medium-anthropomorphism (M = 4.09, SD = 1.03), and low-anthropomorphism (M = 1.79, SD = 0.59) conditions (all Bonferroni-adjusted *p*s < 0.01). Meanwhile, differences among the three images within the high-anthropomorphism set [F(2, 58) = 1.459, *p* = 0.241], within the medium-anthropomorphism set [F(2, 58) = 1.577, *p* = 0.215], and within the low-anthropomorphism set [F(2, 58) = 0.165, *p* = 0.849] were all non-significant.

To test the manipulation of empathic responding, we used the Emotional Responsiveness dimension of the PETS (Perceived Empathy of Technology Scale) as a manipulation-check measure on a 7-point Likert scale [34] (e.g., “The AIMCA took my psychological state into account,” “The AIMCA provided support when I was dealing with an emotional situation”). A paired-samples *t*-test comparing the two response styles showed a significant difference between the empathic and non-empathic conditions [t(29) = 32.736, *p* < 0.001], with higher perceived empathy in the empathic-response condition (M = 5.09, SD = 0.29) than in the non-empathic-response condition (M = 2.61, SD = 0.26). Therefore, the manipulation checks for both anthropomorphic appearance and empathic responding were considered effective.

### 2.2. Participants

An a priori power analysis was conducted in GPower 3.1 to determine the required sample size [35]. Given the 3 × 2 experimental structure and our focus on the interaction between anthropomorphism and empathic responding, we adopted a relatively conservative specification in GPower by selecting F tests → ANOVA: repeated measures, within factors. We set the effect size to a medium level (Cohen’s f = 0.25) [36], with a significance level of α = 0.05, statistical power of 1 − β = 0.90, and assumed a moderate correlation among repeated measures (ρ = 0.30) and sphericity (ε = 1.00). The results indicated that at least N = 74 valid participants were required to detect the expected effects. Considering that eye-tracking studies may require participant exclusion due to insufficient calibration accuracy, loss of gaze, or data-quality issues, we planned to recruit approximately 10–15% more participants beyond the minimum, targeting 82–86 participants to ensure adequate power in the final valid sample.

Participants were recruited via WeChat (a Chinese social media platform) and campus posters. A total of 85 undergraduate and graduate students from Shenzhen Technology University took part in the study. Data from five participants were excluded due to low eye-tracking accuracy. The final sample therefore comprised 80 valid participants (44 male, 36 female), aged 20–29 years (M = 23.63, SD = 2.37). Participants came from a range of majors, including mechanical engineering, computer science, economics and trade, design, and linguistics. All participants were in good health, had normal or corrected-to-normal vision, and reported no color blindness, color weakness, or astigmatism. After the experiment, each participant received RMB 35 or a cup of coffee as compensation.

### 2.3. Ethics Approval and Informed Consent

This study was conducted in accordance with the relevant principles of the Declaration of Helsinki and received approval from the Ethics Review Committee of Shenzhen Technology University (Approval No. SZTU-IRP20250031). Before the formal experiment began, all participants were informed of the purpose of the study, the experimental procedures, the potential risks, and the intended use of the data, and provided written informed consent on a voluntary basis. Participants were informed that they were free to withdraw from the experiment at any stage without any adverse consequences. All data were used solely for academic research and were processed, stored, and analyzed anonymously.

### 2.4. Apparatus

This study used the wearable binocular eye-tracking system Tobii Pro Glasses 3 to record participants’ visual behavior. The device estimates gaze points based on corneal reflection, dark pupil, and stereogeometric principles, and its sampling rate can be set to 50 or 100 Hz; in the present study, it was set to 100 Hz. According to the official product specifications, the average accuracy of the device is approximately 0.6°. The synchronized first-person scene video was recorded at a resolution of 1920 × 1080 with a frame rate of 25 fps. The diagonal field of view was approximately 106°, with horizontal and vertical fields of view of approximately 95° and 63°, respectively [37]. According to Tobii’s publicly available data quality documentation, under controlled test conditions, Tobii Pro Glasses 3 have an average precision of approximately 0.03° and an average data loss rate of approximately 0.01% [38]. Gaze mapping, area-of-interest (AOI) definition, and metric export were performed using Tobii Pro Lab (Version 24.21). Although the present study employs a screen-based stimulus task, the wearable eye tracker allowed gaze data to be collected under relatively natural head postures. To reduce the influence of mapping error on AOI-based measures, viewing distance was controlled and segmented drift checks were conducted during the experiment. The criteria for excluding low-quality eye-tracking data are as follows: if, during the initial calibration or subsequent segmented validation, the average validation error remained above the predefined threshold after recalibration (1.5° in the present study); if a clear and persistent systematic offset was observed near the key AOIs, such that fixation points could not be stably mapped onto the corresponding interface elements; or if the proportion of missing valid gaze samples exceeded 20%, the participant’s eye-tracking data were excluded from the final analyses. These thresholds were determined with reference to the commonly recommended spatial buffer of 1–1.5° in AOI research—with evidence showing that AOI classification errors increase when accuracy exceeds 1.0° and with the common practice in wearable eye-tracking studies of excluding records with substantial data loss [39,40,41].

### 2.5. Procedure

The experiment was conducted in an ergonomics laboratory that was sound-insulated, stably illuminated, and maintained at a comfortable temperature. Upon arrival, each participant first read the study instructions and signed the informed consent form, and was then guided into a standardized scenario: they were asked to imagine that they were using an AIMCA for an online health consultation and to form immediate judgments based on the cues presented on the interface. Participants remained seated throughout the experiment, and viewing distance was controlled at approximately 60–70 cm. To minimize gaze-mapping errors in this screen-based task when using wearable eye tracking, participants were instructed to maintain a relatively stable head position during the experiment and were reminded, when necessary, to return to the standard seated posture. The reporting of the eye-tracking study and the description of data quality followed existing best-practice recommendations and minimum reporting guidelines.

**Figure 1:**
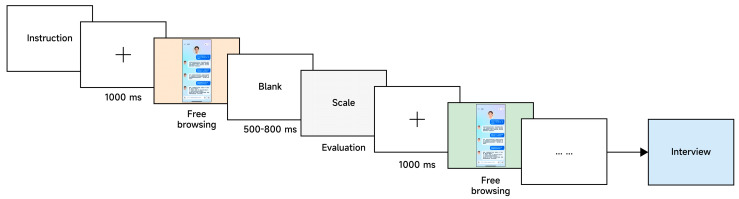
Eye-tracking experimental procedure.

**Figure 2:**
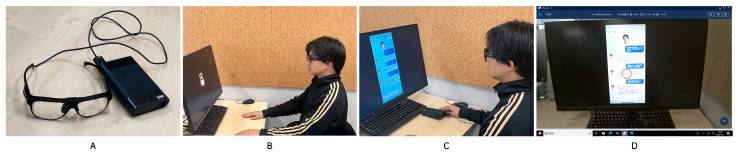
Photographs of the experimental setting. (**A**) the experimental equipment, (**B**) the gaze calibration procedure, (**C**) the experiment in progress, and (**D**) the software interface from the participant’s first-person perspective.

To collect subjective evaluations, eye-tracking data, and qualitative feedback, the experiment consisted of two main steps. Before the formal experiment began, the researcher fitted each participant with the Tobii Pro Glasses 3 and conducted standard calibration and validation; if calibration quality did not meet the required standard, recalibration was repeated until the data collection criteria were satisfied. In the first step, participants freely viewed the current AIMCA interface prototype, with no time limit imposed, in order to capture natural gaze behavior and attentional allocation. In the second step, participants completed a subjective evaluation of that interface at their own pace, thereby providing their immediate impressions of the AIMCA. After the evaluation was completed, the system automatically presented the next interface prototype. To control for order effects, the presentation order of the stimuli was randomized for each participant. After all prototypes had been viewed, the researcher conducted a brief semi-structured interview focusing mainly on participants’ first impressions of appearance anthropomorphism, their perceptions of empathic responses, the basis on which they judged information credibility, and their concerns about potential risks. The interview content was summarized around these themes, and its purpose is to provide contextualized interpretation for key patterns in the quantitative results; accordingly, the relevant interview insights are presented in the Section 4. Each participant took approximately 25–30 min in total. The eye-tracking procedure is shown in Figure 1. The experimental setting and photographic documentation are presented in Figure 2, where A shows the experimental equipment, B the gaze calibration procedure, C the experiment in progress, and D the software interface from the participant’s first-person perspective.

### 2.6. Measures and Eye-Tracking Metrics

In the present study, we focused primarily on users’ subjective perceptions and visual behavior while they freely viewed AIMCAs. The evaluation scales comprised two constructs, namely perceived trust and behavioral intention. All measures were assessed using seven-point Likert scales (1 = strongly disagree, 7 = strongly agree). Perceived trust was measured using an adapted version of the trust in automation scale developed by Jian’s research [42]. This scale has been widely adopted in engineering psychology and human factors research and has long been regarded as one of the representative instruments for assessing human trust in automation or AI systems [43,44]. Behavioral intention was measured using the scale developed by Venkatesh’s research [45]. As a core outcome variable in technology adoption research, the UTAUT measure of behavioral intention has been widely applied in contexts such as health technologies and AI health assistants, thereby supporting comparability with the existing HCI and health informatics literature [46,47].

Further details are provided here regarding the source, item composition, contextual adaptation, and reliability of the two scales in the present sample. The perceived trust scale consists of five items, with a sample item being: “TR1. I believe that the recommendations provided by this AIMCA are generally reliable.” The behavioral intention scale consists of three items, with a sample item being: “BI3. I plan to use this AIMCA in the near future.” Both scales were contextually adapted to the AIMCA setting; specifically, items originally referring to general automation systems or technology use were revised to reflect interface evaluation and usage judgments in relation to AIMCAs. Because the original versions of both scales were in English, a back-translation procedure was employed to maintain cross-language semantic equivalence [48,49]. Specifically, in October 2025, we invited a researcher in applied linguistics to translate the questionnaire into Chinese. We then invited another design researcher with proficiency in both Chinese and English to translate the Chinese version back into English. After that, we met with the researchers to compare the original and back-translated versions and further standardized the wording in light of the research context to ensure that the semantic meaning of the items was appropriate for the medical conversational agent setting. In the present sample, the perceived trust scale showed an internal consistency of Cronbach’s α = 0.887, and the behavioral intention scale showed an internal consistency of Cronbach’s α = 0.893, indicating good internal consistency for both scales in this study. Given the substantial variation in conceptual boundaries and item selection across human–automation trust measures, clearly reporting the source of the scales, the adaptation procedure, and the reliability of the present sample helps improve the comparability and reproducibility of the findings [44,50].

**Figure 3:**
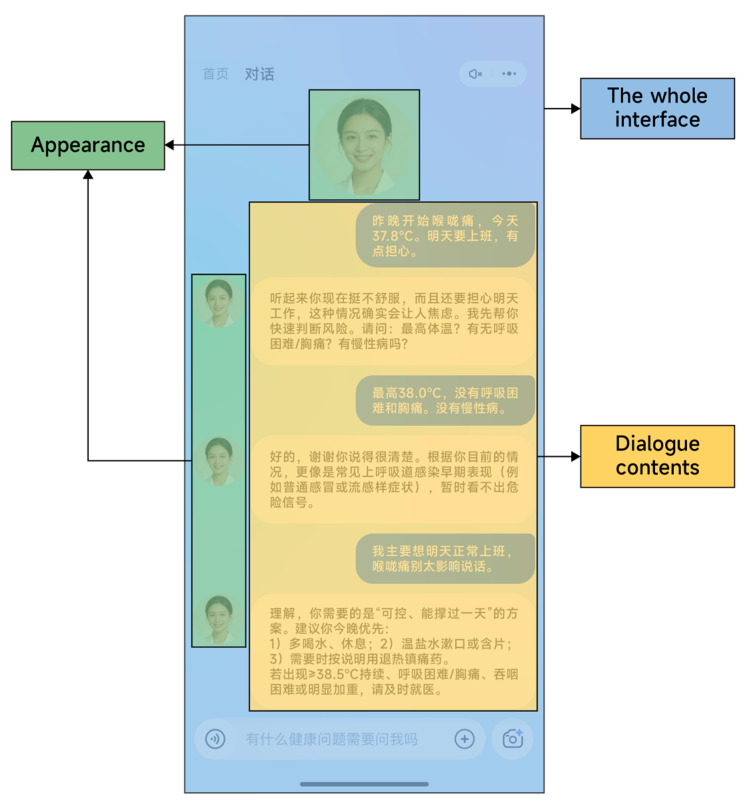
Definition of each AOI on the AIMCA interface.

To analyze the eye-tracking measures, three areas of interest (AOIs) were defined for each trial: the appearance presentation area of the AIMCA, the conversational content area, and the overall interface area. Figure 3 shows the specific AOI delineation.

To improve comparability across different AOIs, the AOI-level eye-tracking measures were adjusted for area on the basis of coverage proportion. Specifically, the coverage proportion of each AOI was first calculated, as shown in Equation (1).

(1) $$p_{AOI}=\frac{{Area}_{AOI}}{{Area}_{Stimulus}}$$

where *Area_AOI_* represents the pixel area of the AOI, *Area_Stimulus_* represents the total pixel area of the corresponding stimulus interface, and *p_AOI_* denotes the coverage proportion of that AOI within the entire stimulus interface. Subsequently, the three AOI measures were standardized separately, as shown in Equations (2)–(4):

(2) $${FC}_{norm}=\frac{{FC}_{raw}}{p_{AOI}}$$

(3) $${MFD}_{norm}=\frac{{MFD}_{raw}}{p_{AOI}}$$

(4) $${DT}_{norm}=\frac{{DT}_{raw}}{p_{AOI}}$$

where *FC_raw_* denotes the raw fixation count, *MFD_raw_* the raw mean fixation duration, and *DT_raw_* the raw dwell time. *FC_norm_*, *MFD_norm_*, and *DT_norm_* represent the fixation count, mean fixation duration, and dwell time, respectively, after adjustment for AOI coverage proportion. This procedure helps reduce the direct influence of differences in AOI on the metric values, so that descriptive comparisons across AOIs can focus more on attentional investment and dwell characteristics rather than on region size itself. Previous methodological research on AOIs has shown that the size, shape, and position of an AOI can directly affect AOI statistics, and that the relevant operationalization procedures therefore need to be clearly reported in the method section. In empirical studies, size effects are also commonly addressed through AOI area control or coverage-based normalization [33,51,52]. Accordingly, all subsequent descriptive statistics reported for AOIs in this study are based on the standardized values defined above.

At the interpretive level, fixation count and dwell time more directly reflect the intensity of attentional investment per unit area, whereas mean fixation duration is used to further characterize dwell features within a given unit area. Placing all three measures within the same AOI coverage-adjustment framework helps maintain a consistent statistical standard at the AOI level and improves the interpretability of cross-AOI comparisons.

## 3. Results

In the present study, perceived trust and behavioral intention were treated as the primary outcome variables, whereas AOI-level eye-tracking measures were treated as process-oriented secondary outcome variables. Accordingly, Section 3.1 is primarily intended to test the subjective outcome hypotheses corresponding to H1a–H3b, while Section 3.2 is primarily intended to address the exploratory research question RQ1 concerning patterns of visual attentional allocation. Both the subjective scale data and the eye-tracking data were analyzed using repeated-measures analysis of variance (repeated-measures ANOVA), with all statistical analyses conducted in SPSS 25.0. This analytical framework was selected for three reasons. First, the study employs a fully balanced 3 (appearance anthropomorphism: high/medium/low) × 2 (empathic response: present/absent) within-subject factorial design. Second, the research questions focus on the main and interaction effects at the condition level. Third, all measures entered the analyses as condition-aggregated observations. Under these circumstances, repeated-measures ANOVA corresponds directly to the theoretical questions of the study and facilitates the reporting of main effects, interaction effects, and their effect sizes [13,25]. Within this analytical framework, the three parallel prototypes in each experimental condition first served the purposes of stimulus sampling and randomized presentation, and were then aggregated at the condition level for the main analyses. Accordingly, the statistical inferences reported in this study are directed primarily toward the effect structure of the two fixed experimental factors, namely appearance anthropomorphism and empathic response.

For the eye-tracking data, repeated-measures ANOVAs were conducted separately for fixation count, mean fixation duration, and dwell time across the three predefined AOIs, in order to characterize how different design cues influence patterns of attentional allocation and dwell behavior. The rationale for analyzing each AOI and each metric separately is that the overall interface area, the appearance area, and the conversational content area are functionally distinct, whereas the three metrics respectively capture frequency of attentional engagement, local dwell characteristics, and overall time investment. Separate analyses therefore helped maintain consistency between AOI interpretation and metric meaning [33]. Unless otherwise specified, both the AOI-level descriptive statistics and the subsequent inferential analyses were based on the area-adjusted measures described in Section 2.5. Accordingly, the M and SD values reported in Section 3.2 are standardized values.

For all effects involving a three-level within-subject factor, sphericity was tested first. When the assumption of sphericity was violated, Greenhouse–Geisser corrections were applied, and the corrected degrees of freedom and significance levels were reported. If any eye-tracking measure showed a markedly skewed distribution, a logarithmic or square-root transformation was applied before analysis to improve normality and homogeneity of variance, after which the same statistical tests were conducted [53].

With regard to multiple comparisons, all post hoc pairwise comparisons were adjusted using the Bonferroni correction. Given that the eye-tracking analyses involved multiple AOIs and multiple outcome measures simultaneously, interpretation focused primarily on the predefined AOI structure, effect size magnitude, and consistent patterns across metrics and regions, in order to enhance the interpretability of the process-oriented findings. Recent eye-tracking studies have likewise commonly adopted Bonferroni or Holm–Bonferroni corrections when handling grouped comparisons, thereby improving the transparency of multiple-comparison reporting [54,55].

In addition, given that each condition in the present study contains multiple prototype instances, mixed-effects models based on trial-level data could provide a more fine-grained way of handling repeated observations within participants and variation across stimulus instances, and are especially suitable when both participants and stimuli are included in the random-effects structure [51]. However, in light of the current sample size, the aims of the study, and the use of condition-level aggregated observations as the unit of analysis, repeated-measures ANOVA was adopted as the primary analytical framework. The value of modeling stimulus-level variability and extending the analyses with mixed-effects models is discussed further in the Limitations section.

An overview of the results is provided in Table 2. Overall, both the anthropomorphic appearance and the empathic response of the AIMCA show significant main effects on users’ perceived trust and behavioral intention, and also formed an interaction effect at the level of subjective outcomes. By contrast, the eye-tracking results primarily reveal partial significant main effects, with no stable interaction pattern observed.

### 3.1. Self-Report Results

Before presenting the analyses of the subjective outcomes, it should be noted that both self-report scales demonstrated good internal consistency in the present sample. Repeated-measures ANOVAs were conducted separately to examine users’ trust in AIMCAs and their behavioral intention across different levels of anthropomorphic appearance (high, medium, and low) and empathic response (present vs. absent). Table 3 presents the means (and standard deviations) of perceived trust and users’ behavioral intention under the different experimental conditions.

**Figure 4:**
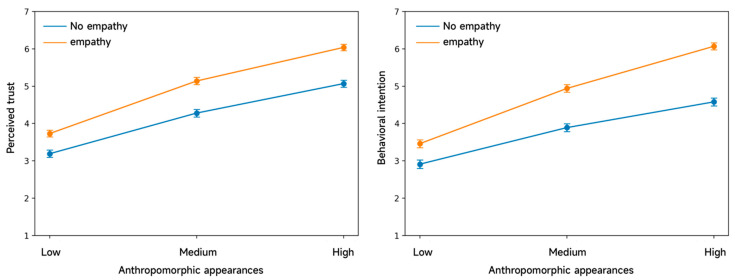
Interaction effects of anthropomorphic appearance and empathic responding on perceived trust and behavioral intention.

The repeated-measures ANOVAs indicate significant main effects of appearance anthropomorphism [F(2, 158) = 726.20, *p* < 0.001, ηp^2^ = 0.902] and empathic responding [F(1, 79) = 226.46, *p* < 0.001, ηp^2^ = 0.741] on users’ perceived trust. In addition, we observed a significant interaction between appearance anthropomorphism and empathic responding [F(2, 158) = 5.42, *p* = 0.005, ηp^2^ = 0.064]. As shown in Table 3, across all appearance conditions, AIMCAs with empathic responses received higher trust ratings than those without empathic responses. Figure 4 illustrates this interaction: empathic responding increased trust at all three levels of appearance anthropomorphism, and the magnitude of this increase grew as the appearance became more anthropomorphic, suggesting an interaction trend whereby stronger anthropomorphic cues yield larger empathy-related trust gains. Post hoc analyses further shows that, under high (*p* < 0.001), medium (*p* < 0.001), and low (*p* < 0.001) levels of appearance anthropomorphism, perceived trust was higher for AIMCAs with empathic responses than for those without. Likewise, within both the empathic (*p* < 0.001) and non-empathic (*p* < 0.001) response conditions, perceived trust follows an ordered pattern, being highest for high anthropomorphism, second-highest for medium anthropomorphism, and lowest for low anthropomorphism. Overall, for the outcome variable of perceived trust, the main effect of anthropomorphic appearance, the main effect of empathic response, and the interaction effect between the two all reached statistical significance, indicating that H1a, H2a, and H3a are all supported.

A similar within-subject repeated-measures ANOVA was conducted for behavioral intention. The results show significant main effects of appearance anthropomorphism [F(2, 158) = 331.30, *p* < 0.001, ηp^2^ = 0.807] and empathic responding [F(1, 79) = 236.60, *p* < 0.001, ηp^2^ = 0.750], as well as a significant interaction between the two factors [F(2, 158) = 10.19, *p* < 0.001, ηp^2^ = 0.114]. As indicated in Table 3, across all appearance conditions, behavioral intention was higher for AIMCAs with empathic responses than for those without. Moreover, within each response condition, behavioral intention was highest under high anthropomorphism, followed by medium anthropomorphism, and lowest under low anthropomorphism. Figure 4 further shows that when the AIMCA’s appearance was more anthropomorphic, users reported higher behavioral intention for empathic than non-empathic AIMCAs, and this difference was more pronounced than under medium or low anthropomorphism. Post hoc analyses similarly confirm that, under high (*p* < 0.001), medium (*p* < 0.001), and low (*p* < 0.001) anthropomorphism, behavioral intention was higher with empathic than without empathic responses. Likewise, within both empathic (*p* < 0.001) and non-empathic (*p* < 0.001) response conditions, behavioral intention follows the same ordered pattern (high > medium > low anthropomorphism). Overall, for the outcome variable of behavioral intention, the main effect of anthropomorphic appearance, the main effect of empathic response, and the interaction effect between the two are likewise statistically significant, indicating that H1b, H2b, and H3b are all supported.

### 3.2. Eye-Tracking Results

To address RQ1, the present study further examines patterns of visual attentional allocation across different AOIs as a function of anthropomorphic appearance and empathic response. Unlike the subjective outcomes, the eye-tracking results were characterized mainly by partial main effects, with no stable interaction effects observed overall. This suggests that the effect structure of these two types of social cues at the level of visual processing does not fully correspond to their synergistic pattern at the level of subjective judgment.

#### 3.2.1. Eye-Tracking Metrics for the Overall Interface Region

**Figure 5:**
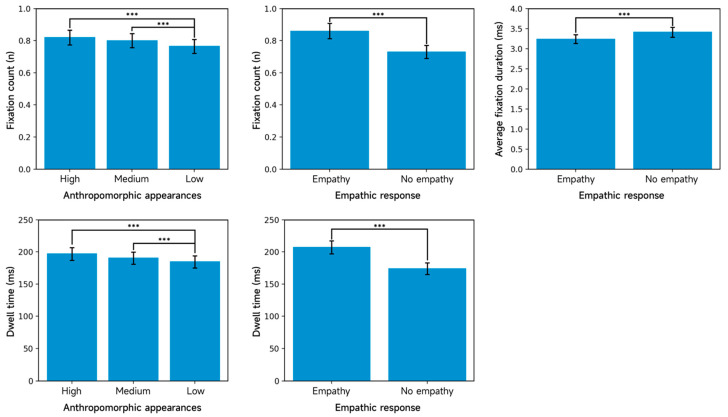
Multiple comparisons of eye-tracking metrics in the overall AIMCA interface region. *** indicates statistical significance at *p* < 0.001.

Table 4 presents the area-adjusted descriptive statistics for the eye-tracking measures in the overall AIMCA interface region. The repeated-measures ANOVA reveals significant main effects of both appearance anthropomorphism and empathic responding on fixation count: the main effect of appearance is significant, F(2, 158) = 46.88, *p* < 0.001, ηp^2^ = 0.372; the main effect of empathic responding is significant, F(1, 79) = 90.69, *p* < 0.001, ηp^2^ = 0.534. No interaction between appearance and empathic responding was observed (*p* > 0.05). Post hoc comparisons of fixation count further clarify the pattern (Figure 5). Highly anthropomorphic AIMCAs (M = 0.820, SD = 0.405) elicited more fixations than low-anthropomorphism AIMCAs (M = 0.765, SD = 0.385, *p* < 0.001), and medium-anthropomorphism AIMCAs (M = 0.800, SD = 0.395) also elicited more fixations than low-anthropomorphism AIMCAs (M = 0.765, SD = 0.385, *p* < 0.001). The difference between the high- and medium-anthropomorphism conditions is not significant (*p* = 0.401). With respect to empathic responding, AIMCAs with empathic responses (M = 0.860, SD = 0.430) attracted more fixations than those without empathic responses (M = 0.730, SD = 0.360, *p* < 0.001).

For mean fixation duration, the repeated-measures ANOVA likewise shows significant main effects of appearance and empathic responding: appearance, F(2, 158) = 30.85, *p* < 0.001, ηp^2^ = 0.281; empathic responding, F(1, 79) = 69.35, *p* < 0.001, ηp^2^ = 0.467. No appearance × empathic responding interaction was observed (*p* > 0.05). Post hoc results are shown in Figure 5. A significant difference emerged between the empathic-response condition (M = 3.240, SD = 0.960) and the non-empathic condition (M = 3.410, SD = 1.083; *p* < 0.001), indicating that empathic responding significantly alters how users process information in the overall interface region, as reflected by mean fixation duration.

For dwell time, the repeated-measures ANOVA again reveals significant main effects of appearance and empathic responding: appearance, F(2, 158) = 11.71, *p* < 0.001, ηp^2^ = 0.129; empathic responding, F(1, 79) = 50.48, *p* < 0.001, ηp^2^ = 0.390. No interaction was observed (*p* > 0.05). Post hoc comparisons of dwell time (Figure 5) shows that highly anthropomorphic AIMCAs (M = 196.850, SD = 87.215) yielded longer dwell time than low-anthropomorphism AIMCAs (M = 184.550, SD = 83.675, *p* < 0.001), and medium-anthropomorphism AIMCAs (M = 190.250, SD = 85.390) also yielded longer dwell time than low-anthropomorphism AIMCAs (M = 184.550, SD = 83.675, *p* < 0.001). The difference between high- and medium-anthropomorphism conditions is not significant (*p* = 0.338). Regarding empathic responding, AIMCAs with empathic responses (M = 207.233, SD = 90.310) elicited longer dwell time than those without empathic responses (M = 173.867, SD = 80.543, *p* < 0.001).

#### 3.2.2. Eye-Tracking Metrics for the Agent Appearance Region

Table 5 presents the area-adjusted descriptive statistics for the eye-tracking measures in the AIMCA appearance region. The repeated-measures ANOVA shows significant main effects of appearance anthropomorphism on all three eye-tracking measures: fixation count, F(2, 158) = 460.19, *p* < 0.001, ηp^2^ = 0.853; mean fixation duration, F(2, 158) = 78.73, *p* < 0.001, ηp^2^ = 0.499; and dwell time, F(2, 158) = 510.83, *p* < 0.001, ηp^2^ = 0.866. In contrast, the main effects of empathic responding were non-significant across all three measures, and the appearance × empathic responding interactions were also non-significant (all *p*s > 0.05).

**Figure 6:**
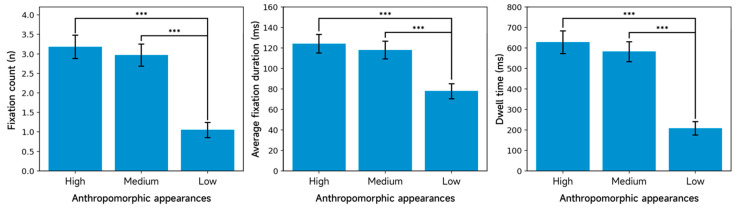
Multiple comparisons of eye-tracking metrics in the AIMCA appearance region. *** indicates statistical significance at *p* < 0.001.

As indicated by the descriptive statistics in Table 5 and the post hoc multiple-comparison plot in Figure 6, fixation count in the high-anthropomorphism condition (M = 3.18, SD = 0.30) is significantly higher than in the low-anthropomorphism condition (M = 1.05, SD = 0.20, *p* < 0.001). Fixation count in the medium-anthropomorphism condition (M = 2.97, SD = 0.29) is also significantly higher than in the low-anthropomorphism condition (M = 1.05, SD = 0.20, *p* < 0.001). However, the difference between the high- and medium-anthropomorphism conditions was not significant (*p* = 0.277). Overall, stronger appearance anthropomorphism elicited more fixations within the appearance AOI, whereas empathic responding did not produce a consistent change in fixation count.

Mean fixation duration was 124.26 ms (SD = 9.12) in the high-anthropomorphism condition, significantly longer than 77.95 ms (SD = 7.34) in the low-anthropomorphism condition (*p* < 0.001). Mean fixation duration was also longer in the medium-anthropomorphism condition (M = 118.10 ms, SD = 8.65) than in the low-anthropomorphism condition (M = 77.95 ms, SD = 7.34, *p* < 0.001). The difference between the high- and medium-anthropomorphism conditions is not significant (*p* = 0.301). Taken together, stronger appearance cues prolonged mean fixation duration within the appearance AOI, whereas empathic responding did not yield a stable benefit on this measure.

Post hoc comparisons of dwell time further clarify the pattern. As shown in Figure 6, dwell time was 628.58 ms (SD = 55.41) in the high-anthropomorphism condition, significantly longer than 208.26 ms (SD = 33.22) in the low-anthropomorphism condition (*p* < 0.001). Dwell time in the medium-anthropomorphism condition (M = 582.47 ms, SD = 48.84) was also significantly longer than in the low-anthropomorphism condition (M = 208.26 ms, SD = 33.22, *p* < 0.001). However, the dwell-time difference between the high- and medium-anthropomorphism conditions is not significant (*p* = 0.075). This pattern indicates that increasing appearance anthropomorphism substantially increased overall gaze dwell within the appearance AOI, whereas empathic responding was insufficient to further amplify this effect.

#### 3.2.3. Eye-Tracking Metrics for the Dialog Content Region

**Figure 7:**
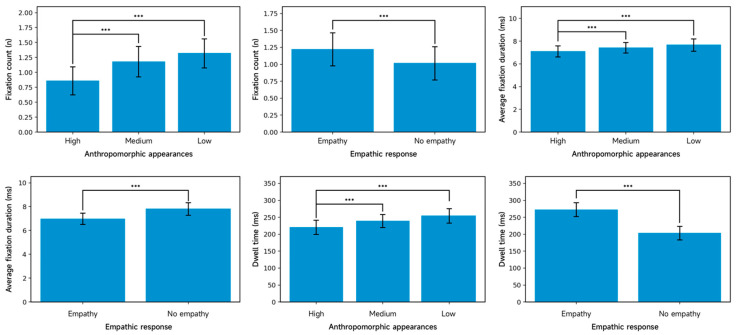
Multiple comparisons of eye-tracking metrics in the AIMCA dialog content region. *** indicates statistical significance at *p* < 0.001.

Table 6 presents the area-adjusted descriptive statistics for the eye-tracking measures in the AIMCA conversational content region. The repeated-measures ANOVA reveals significant main effects of appearance anthropomorphism [F(2, 158) = 103.49, *p* < 0.001, ηp^2^ = 0.567] and empathic responding [F(1, 79) = 85.07, *p* < 0.001, ηp^2^ = 0.518] on fixation count. No interaction effect was observed [F(2, 158) = 1.98, *p* = 0.141]. Post hoc comparisons of fixation count further clarify the pattern (Figure 7). Specifically, low-anthropomorphism AIMCAs (M = 1.32, SD = 0.21) attracted more fixations than high-anthropomorphism AIMCAs (M = 0.86, SD = 0.19, *p* < 0.001), and medium-anthropomorphism AIMCAs (M = 1.18, SD = 0.22) also attracted more fixations than high-anthropomorphism AIMCAs (M = 0.86, SD = 0.19, *p* < 0.001). The difference between the low- and medium-anthropomorphism conditions was not significant (*p* = 0.094). Regarding empathic responding, AIMCAs with empathic responses (M = 1.22, SD = 0.20) elicited more fixations than those without empathic responses (M = 1.02, SD = 0.19, *p* < 0.001).

For mean fixation duration, the repeated-measures ANOVA again shows significant main effects of appearance anthropomorphism [F(2, 158) = 30.11, *p* < 0.001, ηp^2^ = 0.276] and empathic responding [F(1, 79) = 45.25, *p* < 0.001, ηp^2^ = 0.364], with no interaction between the two factors [F(2, 158) = 2.52, *p* = 0.084]. Post hoc comparisons (Figure 7) indicate that mean fixation duration was longer for low-anthropomorphism AIMCAs (M = 7.67, SD = 0.46) than for high-anthropomorphism AIMCAs (M = 7.11, SD = 0.39, *p* < 0.001), and longer for medium-anthropomorphism AIMCAs (M = 7.42, SD = 0.36) than for high-anthropomorphism AIMCAs (M = 7.11, SD = 0.39, *p* < 0.001). The difference between the low- and medium-anthropomorphism conditions is not significant (*p* = 0.591). Regarding empathic responding, mean fixation duration was longer for AIMCAs without empathic responses (M = 7.81, SD = 0.40) than for those with empathic responses (M = 6.98, SD = 0.36, *p* < 0.001).

For dwell time, the repeated-measures ANOVA reveals significant main effects of appearance anthropomorphism [F(2, 158) = 13.92, *p* < 0.001, ηp^2^ = 0.150] and empathic responding [F(1, 79) = 76.12, *p* < 0.001, ηp^2^ = 0.491], with no interaction effect [F(2, 158) = 0.85, *p* = 0.431]. Post hoc comparisons of dwell time (Figure 7) show that dwell time was significantly longer for low-anthropomorphism AIMCAs (M = 254.62, SD = 15.89) than for high-anthropomorphism AIMCAs (M = 220.76, SD = 16.03, *p* < 0.001), and also longer for medium-anthropomorphism AIMCAs (M = 239.34, SD = 15.05) than for high-anthropomorphism AIMCAs (M = 220.76, SD = 16.03, *p* < 0.001). The difference between the low- and medium-anthropomorphism conditions is not significant (*p* = 0.331). Regarding empathic responding, dwell time was longer for AIMCAs with empathic responses (M = 272.94, SD = 14.39) than for those without empathic responses (M = 203.54, SD = 13.77, *p* < 0.001).

## 4. Discussion

To investigate the interactive effects of anthropomorphic appearance and empathic response on users’ subjective perceptions of and visual behavior toward AIMCAs, this study designed AIMCAs with three levels of anthropomorphism and two types of empathic response and used eye-tracking and subjective evaluation methods to record users’ eye-movement measures and perceptions. The results indicate that the anthropomorphic appearance and empathic response of AIMCAs exert both shared and distinct effects on users’ visual behavior and perceptions.

The findings further show that anthropomorphic appearance and empathic response jointly enhanced users’ perceived trust and behavioral intention. By contrast, no such interaction effect was found in users’ visual behavior. AIMCAs with a higher level of anthropomorphism and empathic responses attracted more fixations and longer dwell times. Taken together, these findings suggest that, in the present prototype-viewing task, anthropomorphic appearance and empathic response can jointly shape users’ subjective perceptions of AIMCAs and influence the allocation of their visual attention.

### 4.1. Effects on User Perception

Taken together, the empirical findings indicate that both appearance-based anthropomorphic cues and empathic response cues embedded in AIMCA dialog significantly shape users’ subjective perceptions of the system, particularly their initial trust judgments and short-term intention to use. More importantly, the results suggest that these two types of social cues do not merely produce additive effects, but instead show an enhancing effect. When visual embodiment and empathic response reinforce one another along the two dimensions of “humanlikeness” and “warmth,” users are more likely to interpret the system as an interactive entity with social presence and communicative intent, which in turn enhances their overall judgments of its professionalism, benevolence, and dependability [12]. Brief post-experimental interview feedback provided additional support for this pattern. With respect to first impressions of appearance, participants typically described highly anthropomorphic interfaces as more similar to a real consultation partner and as something they would be more willing to approach or continue exploring. With respect to language style, interfaces with empathic responses were more likely to be experienced as making them “feel understood” or as something they were “more willing to continue reading.” These observations are consistent with the direction of the subjective findings and suggest that appearance cues primarily influence first impressions and approach tendencies, whereas empathic cues more directly enter the interaction climate and the process of content acceptance.

First, regarding the mechanism through which anthropomorphic appearance affects user perception, anthropomorphic appearance is, in essence, a strong social cue. It can activate users’ sense of social presence and personifying attributions, making them more likely to interpret system behavior through an interpersonal framework and, on this basis, form stronger relational trust and higher expectations for communication. Previous studies in health-related settings have shown that anthropomorphic cues can promote acceptance of and continued use intention toward health conversational agents by enhancing social presence and supporting trust formation [12,19]. Accordingly, when an AIMCA has a more anthropomorphic appearance, users are more likely to feel that it is a social entity capable of responding to them and understanding them, which in turn fosters more positive initial trust judgments and increases their willingness to proceed to further information evaluation and usage attempts.

Second, the effect of empathic response on user perception is more directly related to the affective and relational quality of the interaction process. In medical communication, users often experience uncertainty and perceived risk. By acknowledging emotions and expressing understanding and support, empathic responses can reduce the sense of communicative threat and strengthen the feeling of being attended to, thereby significantly improving users’ overall evaluations of information quality and interaction experience. Existing studies support this point through different lines of evidence. In public health question-answering contexts, clinicians’ evaluations of the empathy and overall quality of AI chatbot responses have often been no lower than those of human-generated responses, suggesting that empathy expressed at the linguistic level can itself substantially enhance perceived experience [56]. Further, in response to cancer-related questions, patients may also perceive AI-generated replies as more empathic. At the same time, research has emphasized that such perceived empathy is more likely to arise from linguistic strategies and textual patterns than from any genuine emotional experience [15]. Therefore, empathic response in AIMCAs represents an important communicative cue that shapes users’ initial judgments and short-term acceptance tendencies, especially in medical interaction scenarios that require reassurance, explanation, and companionship.

Third, the interaction pattern between appearance and empathy observed in this study can be explained in terms of cue consistency and expectation confirmation. Anthropomorphic appearance raises users’ expectations that the system will communicate in a humanlike manner, and empathic response then fulfills and reinforces that expectation, thereby producing a more stable overall judgment that the system is trustworthy and usable. By contrast, if the appearance strongly signals sociality while the verbal response lacks empathy and care, users may experience a sense of mismatch in which the system looks human but speaks like a machine, thereby weakening relational trust. In other words, users do not evaluate appearance and language separately; rather, they assess whether the system as a whole presents a coherent persona and interactional intent. Another set of interview responses focused more on judgments such as “whether the information has evidence,” “whether the content is trustworthy,” and “whether I would still verify it further.” These statements suggest that anthropomorphic appearance and empathic response can increase users’ initial acceptance and willingness to continue reading, but that their final judgment of trustworthiness remains closely tied to content evidence, clarity of explanation, and awareness of risk. This supplementary observation corresponds well with the later discussion of trust calibration. It is also consistent with empirical studies in the health conversational agent literature on the pathways linking anthropomorphic cues, social presence, trust, and adoption or continued use [12,19].

Finally, increasing trust should not be equated with maximizing unconditional trust, but should instead be oriented toward trust calibration. In health-related contexts, user perceptions may be elevated by anthropomorphic and empathic cues, but this may also introduce the risk of inappropriate reliance and overestimation of system capability. A recent systematic review on trust in medical AI has shown that the factors shaping trust include not only system transparency, reliability, and safety, but also contextual fit and individual differences. Trust should therefore be designed as a dynamic state that can be supported by evidence and corrected when necessary [57]. At the same time, discussions at the health-system level have emphasized that the integration of AI into healthcare services will reconfigure the trust network among patients, clinicians, institutions, and technologies, and that sustainable trust must be maintained through accountability mechanisms, validation, and governance [58]. Accordingly, in the development of AIMCAs, while pursuing the perceptual benefits brought by anthropomorphism and empathy, designers should also incorporate boundary cues and information structures that are explainable and verifiable, so that users can be guided, through appropriate forms of explanation, toward trust and use decisions that are aligned with the actual capabilities of the system [59].

### 4.2. Effects on Users’ Visual Behavior

From the overall pattern of the eye-tracking results, both anthropomorphic appearance and empathic response significantly altered users’ allocation of attention. However, at the level of visual behavior, these effects were manifested primarily as independent main effects rather than as stable synergistic interaction effects. This pattern is consistent with recent eye-tracking research on anthropomorphic cues in chatbots: although anthropomorphic appearance and humanlike conversational style often produce stronger joint effects in subjective perception, they do not necessarily show interaction structures of comparable strength in visual measures such as fixation count, mean fixation duration, and dwell time [13].

First, the eye-tracking results at the overall interface level suggest that the two types of cues jointly increased users’ visual engagement, although their effects on patterns of visual dwell were not entirely the same. In general, fixation count and dwell time are more directly related to attentional investment and viewing coverage, whereas mean fixation duration more closely reflects the intensity of information processing or momentary cognitive load within a single fixation [60,61]. Therefore, when the results show that empathic response increased overall fixation and dwell while producing a different directional pattern in mean fixation duration, a more cautious interpretation is that empathic response not only attracted more visual attention, but also influenced the way users organized their visual dwelling while browsing the interface. In other words, while maintaining a higher level of engagement, users altered the way they progressed through the information, showing a pattern of visual interaction characterized by sustained involvement but an adjusted rhythm of dwelling.

Second, in the appearance AOI, changes in visual behavior were driven mainly by the strength of appearance anthropomorphism, whereas the effect of empathic response was not significant. This finding is consistent with existing explanations that more humanlike appearances are more likely to attract visual attention: appearances with stronger anthropomorphic features are more likely to become visual objects that users prioritize and continue to inspect, thereby significantly increasing fixation and dwell in the appearance region [62]. At the same time, research on anthropomorphic cues has suggested that certain humanlike features may first influence users’ initial attentional orientation and sustained attention, and may subsequently affect later perceptual and affective processing through attentional allocation [63]. Accordingly, the results for the appearance AOI can be summarized more concisely as follows: anthropomorphic appearance, as a strong visual social cue, preferentially competes for attentional resources and produces stronger visual dwell; by contrast, empathic response is a language-level cue whose primary domain of influence does not lie in the appearance region, and therefore it is less likely to generate stable gains in appearance-AOI measures.

Third, the reversed attentional allocation pattern observed in the conversational content AOI is theoretically important. When appearance anthropomorphism was weaker, users fixated on the text content region more frequently, dwelled there longer, and showed longer mean fixation durations. Some of the brief post-experimental interview feedback revealed a similar tendency: when the social cues embedded in the appearance were weaker, participants were more likely to shift their attention to the text itself and to form judgments around questions such as “I want to further confirm whether the information is sufficient” and “whether this information has a credible basis.” These subjective reports are consistent with the direction of the greater fixation and dwell observed in the content region, and help explain the pattern of enhanced content engagement under low-anthropomorphism conditions. This pattern may be understood as reflecting a higher level of content engagement and more cautious reading. In medical contexts, when users face high uncertainty and potential risk, they are more likely to rely on repeated reading, integration, and verification of the content itself in order to form judgments. When appearance cues are insufficient to support rapid social judgments, attentional resources are more likely to shift back to the textual evidence, thereby producing a higher visual processing load in the content region [60,61]. In other words, “looking more” and “looking longer” at the content region does not necessarily indicate a better experience; it may also indicate that users invested more resources in understanding, comparing, and scrutinizing the information.

In addition, empathic response showed a compound effect in the conversational content AOI: on the one hand, it produced more fixations and longer dwell time; on the other hand, mean fixation duration changed accordingly. This suggests that empathic response does not merely attract attention in a simple sense, but also shapes the social context of the interaction and thereby influences the way users follow the content and sustain their reading engagement. Empathic language can reduce perceived communicative threat and strengthen the sense of being understood, making users more willing to continue reading along with the conversational content and remain engaged. Correspondingly, related eye-tracking research on clinical communication has also found observable links between empathy and gaze behavior, indicating that empathic cues affect not only subjective evaluations but also more fundamental processes of attentional allocation and communication [64]. Therefore, the present study is better interpreted as showing that empathic response enhanced users’ sustained engagement with the conversational content and influenced the organization of their information reading and scrutiny.

Finally, the visual behavior findings jointly point to a design principle with clear practical relevance. The anthropomorphic appearance of AIMCAs is better suited to function as an entry cue for establishing a sense of presence and initiating interaction, but it should avoid excessively consuming attentional resources that ought to support content comprehension. By contrast, empathic response is better suited to function as a language-level cue for maintaining interactive engagement and supporting attention to content, and should be combined with structured information presentation, such as segmentation of key points, risk reminders, action recommendations, and verifiable evidence, to ensure that users’ attention ultimately falls on the core information relevant to decision making. Similar patterns of multi-cue attentional allocation have also been observed in research on anthropomorphism and multimodal interaction: different anthropomorphic channels often serve different attentional functions, and truly effective combinations do not simply intensify “human likeness,” but instead enable different cues to work together in support of the task goal [13,63].

### 4.3. Integrative Measurement of Anthropomorphic Appearance and Empathic Responding

The value of an integrated measurement approach lies in linking global outcome variables with process-oriented evidence, thereby allowing a more fine-grained account of how different design cues enter the formation of user judgments. In the present study, anthropomorphic appearance and empathic response showed a clear joint enhancement at the levels of trust and behavioral intention, whereas at the eye-tracking level they were reflected primarily in differences in attentional allocation across different AOIs. This suggests a hierarchical distinction between users’ overall judgments and their immediate visual processes: the former is more closely related to an integrated evaluation of interface appearance, linguistic style, and medical context, whereas the latter reveals the specific ways in which these cues enter and are dwelled upon during browsing. Consistent with findings from related research on conversational agents, this pattern of “stronger subjective integration but more region-specific visual differentiation” helps explain that different social cues do not serve exactly the same function in judgment formation [13,65].

This divergence, whereby subjective judgments are more readily integrated while eye-tracking patterns are more functionally differentiated, is not contradictory; rather, it provides important boundary conditions for understanding the underlying mechanism. Eye-tracking measures more directly reflect the moment-to-moment allocation of attentional resources and are constrained by interface salience, reading strategies, and task goals. Subjective evaluations, by contrast, represent users’ overall inferences about the coherence of the system’s role, its benevolence, and its dependability after integrating appearance cues with the dialog experience. Accordingly, when anthropomorphic appearance strengthens the frame of the system as a social actor, empathic response is more likely to be interpreted as care and understanding rather than as a scripted strategy, thereby producing stronger trust and adoption tendencies at the attitudinal level. At the visual level, however, empathy does not necessarily redirect attention back to the appearance region; instead, it is more likely to influence processing strategies in the content region by altering the rhythm of dialog comprehension and the way risk is evaluated. This interpretation is consistent with the pathways identified in health conversational agent research linking anthropomorphism, social presence, trust, and adoption [12,19].

From a methodological perspective, integrated measurement also allows the discussion to move beyond trust enhancement toward trust calibration. Trust scales themselves are characterized by construct diffusion and a proliferation of measurement tools, such that a single self-report indicator cannot readily distinguish genuine reliance from merely expressed trust [50]. At the same time, the use of eye-tracking to assess trust does not reflect a stable or monotonic relationship; depending on the task and interface, attentional measures may show different directional patterns or only weak associations with trust. Eye-tracking is therefore better suited as a process-oriented complement to subjective judgments rather than as a substitute for them [66]. In this sense, the integrated measurement framework adopted in the present study not only reveals how anthropomorphic appearance and empathic response shape users’ subjective judgments and visual processes, but also provides a diagnostic perspective for design iteration. When subjective trust increases but attention to key content regions remains insufficient, boundary cues and verifiable information structures can be further strengthened. When attention to the content region is relatively high but evaluations remain cautious, information organization and explanatory support can be optimized to reduce comprehension costs and promote the formation of trust that is appropriately aligned with system capabilities.

## 5. Significance and Limitations

### 5.1. Significance

The present study focuses on two key social cues in AIMCAs that can be intentionally designed, namely anthropomorphic appearance and empathic response, thereby addressing a limitation of prior research, which has often examined these two types of cues separately and has relied mainly on self-report scales, making it difficult to capture processes of attentional allocation and information processing. In the high-risk and high-uncertainty context of healthcare, this integrated chain of evidence—from design cues, to perceptual judgments, to processing mechanisms—helps explain how users form initial trust judgments, short-term intention to use, and their possible boundaries and biases when evaluating interface prototypes.

At the theoretical level, this study systematically examines the effect structure of anthropomorphic appearance and empathic response on trust, behavioral intention, and visual attention from the combined perspective of appearance cues and communication cues. The findings show that these two types of social cues can produce clear joint gains at the level of subjective evaluation, while displaying different functional emphases across regions at the visual level. These results extend our understanding of the AIMCA evaluation process by enabling researchers to capture both overall judgment outcomes and visual allocation processes, and thus to more fully explain how users form acceptance judgments about such systems in medical contexts characterized by risk and uncertainty.

At the methodological level, this study adopts a controlled within-subject experiment and combined subjective scales with eye-tracking measures. This approach made it possible not only to test trust and behavioral intention as outcome variables, but also to provide process-oriented evidence at the AOI level, thereby reducing the limitations of relying solely on self-report data when interpreting attentional allocation and information processing. At the same time, the use of static prototypes ensured standardized visual presentation and stable AOI definition, making the findings easier to reproduce and facilitating comparative validation in future studies across different interfaces and tasks.

At the practical level, this study provides a more actionable design basis for levels of anthropomorphic appearance and strategies for empathic response in AIMCAs. On the one hand, anthropomorphic appearance can serve as a strong visual cue that shapes users’ first impressions and guides their entry point of attention. On the other hand, empathic response can function as a key communicative cue that improves interaction experience and trust judgments. More importantly, these two types of cues need to remain consistent in visual presentation and linguistic style in order to reduce incongruity and inappropriate expectations, and to support initial trust judgments and short-term usage decisions that are appropriately aligned with system capabilities.

In summary, the significance of this study lies not only in identifying which design cues are more likely to elicit positive evaluations, but also in providing an evaluation framework that can support iterative design. By examining subjective perceptual outcomes and visual process evidence in parallel, the study contributes to both usability optimization and trust calibration, and provides a stronger human factors and interaction-based evidence foundation for subsequent system validation and design iteration in more realistic medical contexts.

### 5.2. Limitations and Future Research Directions

The present study is subject to several limitations. First, the sample consisted mainly of undergraduate and graduate students from a single university and therefore represents a relatively young group with a concentrated age range, relatively high educational attainment, and broadly similar experience with digital technologies. Although such a sample helps reduce additional variance arising from background differences under controlled experimental conditions, it also means that the present findings are better interpreted as evidence of early-stage judgments made by younger and more highly educated users in prototype-viewing and short-term evaluative contexts. By contrast, potential AIMCA users are more diverse in terms of age, health status, disease severity, digital literacy, and prior experience with technology, and these differences may themselves shape how users judge anthropomorphic appearance, empathic response, information credibility, and usage risk. Actual users of health chat agents span different health tasks and different population groups, and older adults’ acceptance of health chat agents and AI health technologies may also be influenced by factors such as technology anxiety, physical condition, facilitating conditions, and prior experience [4,43]. Therefore, the applicability of the present findings to older adults, people engaged in chronic disease management, or users with low digital literacy requires further examination.

Second, the experimental stimuli consisted of static interface prototypes and predetermined dialog content, and thus represented a short-term, non-interactive evaluation based on hypothetical consultation scenarios. This design was helpful for standardizing visual presentation and stabilizing AOI definition in eye-tracking analysis, thereby allowing clearer identification of the prototype-level effects of the two social cues examined here, namely anthropomorphic appearance and empathic response. At the same time, however, it could not fully capture several key characteristics of real AIMCA interaction, such as multi-turn questioning and clarification, contextual memory, real-time adaptation to user input, error repair, changes in emotion over the course of the dialog, and the sense of engagement generated by response timing and interaction continuity. Real generative medical conversational systems and voice agents can support more context-sensitive real-time interaction, and users’ relational experience and engagement during continued use are likely to be jointly shaped by emotional resonance, sense of safety, goal alignment, and the interaction process itself [5,67]. At the same time, research on empathic agent prototypes has suggested that users may themselves experience some difficulty in recognizing the degree of empathy embodied in prototypes, indicating that the perception of empathy in static or prototyped settings may not be fully equivalent to the experience of empathy in real use [68]. Accordingly, the present findings are better understood as early evidence regarding interface prototype viewing, first-impression formation, and short-term evaluation. They can effectively reveal how anthropomorphic appearance and empathic response influence users’ initial judgments, but further validation in runnable systems and more natural interaction tasks is still needed to explain how empathy, trust, and engagement evolve over the course of sustained dialog in real interactive systems.

Third, trust and behavioral intention are subjective judgments. Although they reflect users’ attitudes, they cannot be directly equated with actual reliance and behavioral adoption, especially in medical contexts, where increased trust may be accompanied by risks of capability overestimation and automation bias [59].

The eye-tracking measures also require cautious interpretation. Although wearable eye tracking is advantageous in screen-based stimulus tasks because it allows participants to maintain a relatively natural posture, gaze mapping and AOI definition may still be affected by head movements, drift, and the density of interface elements, which may reduce the sensitivity of some measures to subtle differences. At the same time, this study primarily employs classical indicators such as fixation count, mean fixation duration, and dwell time. Although these measures can reflect attentional investment and processing rhythm, they still leave room for interpretation with regard to finer-grained cognitive strategies. For example, longer viewing time may indicate greater engagement, but it may also reflect higher comprehension costs or a stronger motivation to verify risk-related information [25,26].

In addition, the main analyses in this study were based primarily on condition-level repeated-measures ANOVA. This strategy is appropriate for addressing questions about main and interaction effects under a 3 × 2 within-subject factorial manipulation, and also facilitates comparability with existing eye-tracking studies on chat agents in terms of result presentation and effect-size reporting [13]. At the same time, three similar prototypes were created for each anthropomorphism level, which helped reduce the incidental influence of any single interface instance on the results and allowed condition effects to be estimated on the basis of a broader set of stimuli. Given the present study’s research aims and unit of analysis, all subjective evaluations and eye-tracking measures were aggregated and analyzed primarily at the condition level with respect to the two experimental factors, appearance anthropomorphism and empathic response. Accordingly, the present findings primarily support the effect structure of these two types of design cues at the condition level, whereas the estimation of differences at the level of individual stimulus instances still leaves room for further refinement. At the same time, because the eye-tracking analyses involved multiple AOIs and multiple outcome measures, and because each condition contained multiple parallel prototype instances, future research could build on trial-level data and further employ linear or generalized linear mixed-effects models, incorporating both participants and stimulus instances into the random-effects structure and re-examining the robustness of the findings within a more unified framework for multiple-testing control [33,51].

Future research may proceed in three directions. First, external validity and applicability should be strengthened by including more representative user groups and health tasks that more closely resemble real use, including participants differing in age, health anxiety, disease severity, and AI literacy, and by conducting follow-up validation in real online consultations or semi-naturalistic settings in order to test the stability and boundary conditions of the findings in high-risk decision contexts [7,11]. Second, interaction realism should be enhanced by extending prototype evaluation to runnable systems and multi-turn dialog, and by taking into account the uncertainty and error risks of generative models in real contexts, so as to further examine how the effects of anthropomorphic appearance and empathic response change during long-term use and how they influence trust recovery and reliance adjustment when errors occur [6]. Third, trust calibration and safety-oriented indicators should be strengthened. In addition to attitudinal scales, future studies should incorporate objective behavioral measures and decision-quality indicators, such as information comprehension and memory, risk recognition, recommendation adoption, and verification behavior, and should test whether interventions such as boundary cues, the presentation of verifiable evidence, and different explanatory strategies can simultaneously improve user experience and reduce automation bias, thereby supporting safer and more sustainable use of medical AI [59].

## 6. Conclusions

This study systematically examines the effects of two social cues in AIMCAs—anthropomorphic appearance and empathic response—on user perception and visual attention by combining subjective evaluation with eye-tracking.

The findings indicate that, in a static prototype-viewing task based on hypothetical consultation scenarios, both anthropomorphic appearance and empathic response improve users’ subjective experience and enhance their initial trust judgments and short-term intention to use. These two types of cues show a synergistic tendency at the level of subjective perception, suggesting that when visual presentation and linguistic style are socially consistent, users are more likely to form a coherent role impression and a more favorable overall judgment.

At the level of visual behavior, anthropomorphic appearance and empathic response exhibited different patterns of attentional allocation. Anthropomorphic appearance more strongly influenced users’ visual dwell in the appearance region, whereas empathic response was more closely associated with sustained attention to the overall interface and the conversational content. By integrating subjective perceptual outcomes with eye-tracking evidence, this study not only identifies which design cues are more likely to elicit positive judgments during prototype evaluation, but also further shows how such judgments unfold in the visual process.

Based on these findings, the design of AIMCAs should address both appearance cues and conversational strategies from the early prototype stage, with particular emphasis on consistency and moderation. Anthropomorphic appearance is well suited to establishing a sense of presence and initiating interaction, whereas empathic response is better suited to reducing perceived communicative threat and enhancing the feeling of being understood. At the same time, combining these cues with boundary prompts, risk disclosure, and verifiable information structures may help optimize users’ interface comprehension, initial judgments, and short-term intention to use during prototype browsing and brief evaluation, while also providing a design basis for subsequent system validation in more realistic interactive contexts.

## Figures and Tables

**Table 1:** Experimental prototypes.

No.	Appearance Anthropomorphism	Empathic Responding	No.	Appearance Anthropomorphism	Empathic Responding
1	High #1	Present	10	High #2	Absent
2	Medium #1	Present	11	Medium #2	Absent
3	Low #1	Present	12	Low #2	Absent
4	High #1	Absent	13	High #3	Present
5	Medium #1	Absent	14	Medium #3	Present
6	Low #1	Absent	15	Low #3	Present
7	High #2	Present	16	High #3	Absent
8	Medium #2	Present	17	Medium #3	Absent
9	Low #2	Present	18	Low #3	Absent

**Table 2:** Summary of measurement results.

Measured Outcomes		Effect	F	*p*	ηp^2^
Subjective outcomes	Perceived trust	AIMCA appearance	726.20	0.000	0.902
		Empathic responding	226.46	0.000	0.741
		AIMCA appearance × Empathic responding	5.42	0.005	0.064
	Behavioral intention	AIMCA appearance	331.30	0.000	0.807
		Empathic responding	236.60	0.000	0.750
		AIMCA appearance × Empathic responding	10.19	0.000	0.114
Eye-tracking outcomes	Fixation count on the overall AIMCA interface	AIMCA appearance	46.88	0.000	0.372
		Empathic responding	90.69	0.000	0.534
		AIMCA appearance × Empathic responding	1.84	0.162	0.023
	Mean fixation duration on the overall AIMCA interface	AIMCA appearance	30.85	0.000	0.281
		Empathic responding	69.35	0.000	0.467
		AIMCA appearance × Empathic responding	2.99	0.053	0.036
	Dwell time on the overall AIMCA interface	AIMCA appearance	11.71	0.000	0.129
		Empathic responding	50.48	0.000	0.390
		AIMCA appearance × Empathic responding	0.74	0.480	0.009
	Fixation count in the AIMCA appearance region	AIMCA appearance	460.19	0.000	0.853
		Empathic responding	0.39	0.535	0.005
		AIMCA appearance × Empathic responding	1.37	0.258	0.017
	Mean fixation duration in the AIMCA appearance region	AIMCA appearance	78.73	0.000	0.499
		Empathic responding	1.25	0.267	0.016
		AIMCA appearance × Empathic responding	0.17	0.846	0.002
	Dwell time in the AIMCA appearance region	AIMCA appearance	510.83	0.000	0.866
		Empathic responding	0.31	0.579	0.004
		AIMCA appearance × Empathic responding	1.31	0.273	0.016
	Fixation count in the AIMCA dialog -content region	AIMCA appearance	103.49	0.000	0.567
		Empathic responding	85.07	0.000	0.518
		AIMCA appearance × Empathic responding	1.98	0.141	0.024
	Mean fixation duration in the AIMCA dialog -content region	AIMCA appearance	30.11	0.000	0.276
		Empathic responding	45.25	0.000	0.364
		AIMCA appearance × Empathic responding	2.52	0.084	0.031
	Dwell time in the AIMCA dialog -content region	AIMCA appearance	13.92	0.000	0.150
		Empathic responding	76.12	0.000	0.491
		AIMCA appearance × Empathic responding	0.85	0.431	0.011

Note. Both the subjective outcomes and the eye-tracking outcomes were analyzed using within-subject repeated-measures ANOVA. For the eye-tracking analyses, separate tests were conducted for the three AOIs and the three metrics, and all post hoc pairwise comparisons were adjusted using the Bonferroni correction.

**Table 3:** Descriptive statistics for perceived trust and behavioral intention.

Condition		Perceived Trust	Behavioral Intention
Appearance Anthropomorphism	Empathic Responding	M (SD)	M (SD)
High	Present	6.04(0.78)	6.07(0.81)
High	Absent	5.07(0.85)	4.58(0.94)
Medium	Present	5.14(0.88)	4.94(0.91)
Medium	Absent	4.28(0.92)	3.89(0.96)
Low	Present	3.73(0.81)	3.46(0.95)
Low	Absent	3.19(0.87)	2.91(0.99)

**Table 4:** Descriptive statistics for eye-tracking metrics in the overall AIMCA interface region.

Condition		Standardized Fixation Count (n)	Standardized Mean Fixation Duration (ms)	Standardized Dwell Time (ms)
Appearance Anthropomorphism	Empathic Responding	M (SD)	M (SD)	M (SD)
High	Present	0.88 (0.44)	3.20 (0.94)	212.40 (92.32)
High	Absent	0.76 (0.37)	3.37 (1.05)	181.30 (82.11)
Medium	Present	0.86 (0.43)	3.24 (0.96)	205.60 (90.19)
Medium	Absent	0.74 (0.36)	3.41 (1.08)	174.90 (80.59)
Low	Present	0.84 (0.42)	3.28 (0.98)	203.70 (88.42)
Low	Absent	0.69 (0.35)	3.45 (1.12)	165.40 (78.93)

Note. All M (SD) values reported in the table are standardized values adjusted for area on the basis of AOI coverage proportion. AOI coverage proportion was defined as the proportion of the total stimulus area occupied by the AOI. The standardization formula is: standardized measure = raw measure ÷ AOI coverage proportion. All three measures were processed using the same formula.

**Table 5:** Descriptive statistics for eye-tracking metrics in the AIMCA appearance region.

Condition		Standardized Fixation Count (n)	Standardized Mean Fixation Duration (ms)	Standardized Dwell Time (ms)
Appearance Anthropomorphism	Empathic Responding	M (SD)	M (SD)	M (SD)
High	Present	3.27 (0.31)	125.84 (9.27)	641.73 (57.18)
High	Absent	3.09 (0.29)	122.67 (8.96)	615.42 (53.64)
Medium	Present	3.03 (0.30)	118.93 (8.41)	593.58 (49.92)
Medium	Absent	2.91 (0.27)	117.26 (8.88)	571.36 (47.75)
Low	Present	1.12 (0.20)	79.31 (7.48)	218.69 (34.57)
Low	Absent	0.98 (0.19)	76.58 (7.19)	197.83 (31.86)

Note. All M (SD) values reported in the table are standardized values adjusted for area on the basis of AOI coverage proportion. AOI coverage proportion is defined as the proportion of the total stimulus area occupied by the AOI. The standardization formula is: standardized measure = raw measure ÷ AOI coverage proportion. All three measures were processed using the same formula.

**Table 6:** Descriptive statistics for eye-tracking metrics in the AIMCA dialog content region.

**Condition**		**Standardized Fixation Count (n)**	**Standardized Mean Fixation Duration (ms)**	**Standardized Dwell Time (ms)**
**Appearance Anthropomorphism**	**Empathic Responding**	**M (SD)**	**M (SD)**	**M (SD)**
High	Present	0.97 (0.22)	6.68 (0.46)	255.76 (21.29)
High	Absent	0.75 (0.25)	7.53 (0.50)	185.77 (19.99)
Medium	Present	1.28 (0.27)	7.04 (0.40)	272.22 (18.21)
Medium	Absent	1.08 (0.24)	7.80 (0.52)	206.46 (20.28)
Low	Present	1.42 (0.24)	7.23 (0.54)	290.83 (22.21)
Low	Absent	1.22 (0.25)	8.11 (0.56)	218.40 (20.44)

Note. All M (SD) values reported in the table are standardized values adjusted for area on the basis of AOI coverage proportion. AOI coverage proportion is defined as the proportion of the total stimulus area occupied by the AOI. The standardization formula is: standardized measure = raw measure ÷ AOI coverage proportion. All three measures were processed using the same formula.

**Figure A1:**
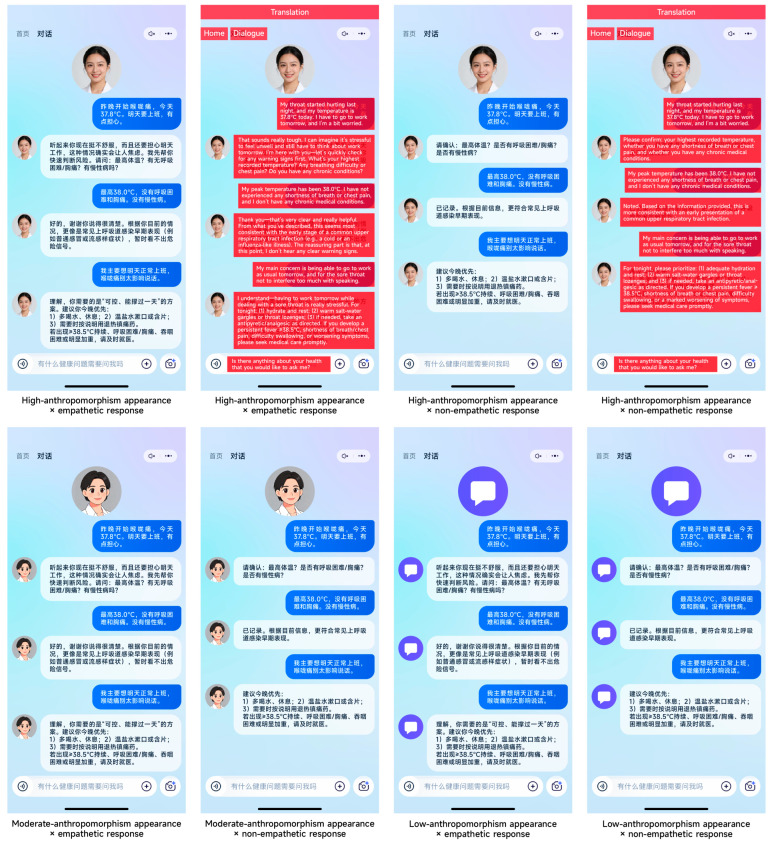
Example of the experimental stimuli.

## Data Availability

The data presented in this study are available upon request from the corresponding author to protect the confidentiality of the participants.

## References

[1] Diederich S., Brendel A.B., Morana S., Kolbe L. (2022). On the design of and interaction with conversational agents: An organizing and assessing review of human-computer interaction research. J. Assoc. Inf. Syst..

[2] Wilson L., Marasoiu M. (2022). The development and use of chatbots in public health: Scoping review. JMIR Hum. Factors.

[3] Ding H., Simmich J., Vaezipour A., Andrews N., Russell T. (2024). Evaluation framework for conversational agents with artificial intelligence in health interventions: A systematic scoping review. J. Am. Med. Inform. Assoc..

[4] Laymouna M., Ma Y., Lessard D., Schuster T., Engler K., Lebouché B. (2024). Roles, users, benefits, and limitations of chatbots in health care: Rapid review. J. Med. Internet Res..

[5] Adams S.J., Acosta J.N., Rajpurkar P. (2025). How generative AI voice agents will transform medicine. Npj Digit. Med..

[6] Huo B., Boyle A., Marfo N., Tangamornsuksan W., Steen J.P., McKechnie T., Lee Y., Mayol J., Antoniou S.A., Thirunavukarasu A.J. (2025). Large language models for chatbot health advice studies: A systematic review. JAMA Netw. Open.

[7] Nong P., Platt J. (2025). Patients’ trust in health systems to use artificial intelligence. JAMA Netw. Open.

[8] Hu X., Gu Y., Lee H.W., Chen X., Li Y., Li X., Zhao Q., Wang W., Huang H., Wang L. (2025). A national survey on the integration of traditional Chinese medicine and artificial intelligence: Attitudes and perceptions from the individuals with health needs. Integr. Med. Res..

[9] Zhang L., Yang J., Fang G. (2025). Factors influencing the acceptance of medical AI chat assistants among healthcare professionals and patients: A survey-based study in China. Front. Public Health.

[10] Hassan M., Kushniruk A., Borycki E. (2024). Barriers to and facilitators of artificial intelligence adoption in health care: Scoping review. JMIR Hum. Factors.

[11] Shevtsova D., Ahmed A., Boot I.W., Sanges C., Hudecek M., Jacobs J.J., Hort S., Vrijhoef H.J.M. (2024). Trust in and acceptance of artificial intelligence applications in medicine: Mixed methods study. JMIR Hum. Factors.

[12] Li Q., Luximon Y., Zhang J. (2023). The influence of anthropomorphic cues on patients’ perceived anthropomorphism, social presence, trust building, and acceptance of health care conversational agents: Within-subject web-based experiment. J. Med. Internet Res..

[13] Chen J., Guo F., Ren Z., Li M., Ham J. (2024). Effects of anthropomorphic design cues of chatbots on users’ perception and visual behaviors. Int. J. Hum.-Comput. Interact..

[14] Seitz L. (2024). Artificial empathy in healthcare chatbots: Does it feel authentic?. Comput. Hum. Behav. Artif. Hum..

[15] Chen D., Chauhan K., Parsa R., Liu Z.A., Liu F.F., Mak E., Eng L., Hannon B.L., Croke J., Hope A. (2025). Patient perceptions of empathy in physician and artificial intelligence chatbot responses to patient questions about cancer. Npj Digit. Med..

[16] Howcroft A., Bennett-Weston A., Khan A., Griffiths J., Gay S., Howick J. (2025). AI chatbots versus human healthcare professionals: A systematic review and meta-analysis of empathy in patient care. Br. Med. Bull..

[17] Ruben M.A., Blanch-Hartigan D., Hall J.A. (2025). What is artificial intelligence (AI) “empathy”? A study comparing ChatGPT and physician responses on an online forum. J. Gen. Intern. Med..

[18] Kohn S.C., De Visser E.J., Wiese E., Lee Y.C., Shaw T.H. (2021). Measurement of trust in automation: A narrative review and reference guide. Front. Psychol..

[19] Liu W., Jiang M., Li W., Mou J. (2024). How does the anthropomorphism of AI chatbots facilitate users’ reuse intention in online health consultation services? The moderating role of disease severity. Technol. Forecast. Soc. Change.

[20] Ng S.W.T., Zhang R. (2025). Trust in AI chatbots: A systematic review. Telemat. Inform..

[21] Afroogh S., Akbari A., Malone E., Kargar M., Alambeigi H. (2024). Trust in AI: Progress, challenges, and future directions. Humanit. Soc. Sci. Commun..

[22] Hagger M.S., Hamilton K. (2025). Progress on theory of planned behavior research: Advances in research synthesis and agenda for future research. J. Behav. Med..

[23] Choudhury A., Shamszare H. (2023). Investigating the impact of user trust on the adoption and use of ChatGPT: Survey analysis. J. Med. Internet Res..

[24] Conner M., Norman P. (2022). Understanding the intention-behavior gap: The role of intention strength. Front. Psychol..

[25] Hooge I.T., Nuthmann A., Nyström M., Niehorster D.C., Holleman G.A., Andersson R., Hessels R.S. (2025). The fundamentals of eye tracking part 2: From research question to operationalization. Behav. Res. Methods.

[26] Valtakari N.V., Hooge I.T., Viktorsson C., Nyström P., Falck-Ytter T., Hessels R.S. (2021). Eye tracking in human interaction: Possibilities and limitations. Behav. Res. Methods.

[27] Huang M., Ki E.J. (2024). Examining the effect of anthropomorphic design cues on healthcare chatbots acceptance and organization-public relationships: Trust in a warm human vs. a competent machine. Int. J. Hum.-Comput. Interact..

[28] Zogaj A., Mähner P.M., Yang L., Tscheulin D.K. (2023). It’s a match! The effects of chatbot anthropomorphization and chatbot gender on consumer behavior. J. Bus. Res..

[29] Lee S.K., Park H., Kim S.Y. (2024). Gender and task effects of human–machine communication on trusting a Korean intelligent virtual assistant. Behav. Inf. Technol..

[30] Aumüller A., Winklbauer A., Schreibmaier B., Batinic B., Mara M. (2024). Rethinking feminized service bots: User responses to abstract and gender-ambiguous chatbot avatars in a large-scale interaction study. Pers. Ubiquitous Comput..

[31] Jin E., Ryoo Y., Kim W., Song Y.G. (2025). Bridging the health literacy gap through AI chatbot design: The impact of gender and doctor cues on chatbot trust and acceptance. Internet Res..

[32] Cummings J.J., Reeves B. (2022). Stimulus sampling and research integrity. Research Integrity: Best Practices for the Social and Behavioral Sciences.

[33] Hooge I.T., Nyström M., Niehorster D.C., Andersson R., Foulsham T., Nuthmann A., Hessels R.S. (2026). The fundamentals of eye tracking part 6: Working with areas of interest. Behav. Res. Methods.

[34] Schmidmaier M., Rupp J., Cvetanova D., Mayer S. Perceived Empathy of Technology Scale (PETS): Measuring empathy of systems toward the user. Proceedings of the CHI Conference on Human Factors in Computing Systems.

[35] Faul F., Erdfelder E., Buchner A., Lang A.G. (2009). Statistical power analyses using G*Power 3.1: Tests for correlation and regression analyses. Behav. Res. Methods.

[36] Cohen J. (2013). Statistical Power Analysis for the Behavioral Sciences.

[37] Tobii Tobii Pro Glasses 3. https://www.tobii.com/products/eye-trackers/wearables/tobii-pro-glasses-3.

[38] Tobii Why Choose Tobii Pro Glasses 3 for Eye Tracking Research. https://www.tobii.com/resource-center/learn-articles/why-choose-tobii-pro-glasses-3-for-eye-tracking-research.

[39] Vehlen A., Standard W., Domes G. (2022). How to choose the size of facial areas of interest in interactive eye tracking. PLoS ONE.

[40] Faraji Y., van Rijn J.W., van Nispen R.M., van Rens G.H., Melis-Dankers B.J., Koopman J., van Rijn L.J. (2023). A toolkit for wide-screen dynamic area of interest measurements using the Pupil Labs Core Eye Tracker. Behav. Res. Methods.

[41] Hessels R.S., Benjamins J.S., Niehorster D.C., van Doorn A.J., Koenderink J.J., Holleman G.A., de Kloe Y.J.R., Valtakari N.V., van Hal S., Hooge I.T.C. (2022). Eye contact avoidance in crowds: A large wearable eye-tracking study. Atten. Percept. Psychophys..

[42] Jian J.Y., Bisantz A.M., Drury C.G. (2000). Foundations for an empirically determined scale of trust in automated systems. Int. J. Cogn. Ergon..

[43] Wong K.K., Han Y., Cai Y., Ouyang W., Du H., Liu C. (2025). From trust in automation to trust in AI in healthcare: A 30-year longitudinal review and an interdisciplinary framework. Bioengineering.

[44] Schlicker N., Baum K., Uhde A., Sterz S., Hirsch M.C., Langer M. (2025). How do we assess the trustworthiness of AI? Introducing the trustworthiness assessment model (TrAM). Comput. Hum. Behav..

[45] Venkatesh V., Morris M.G., Davis G.B., Davis F.D. (2003). User acceptance of information technology: Toward a unified view. MIS Q..

[46] Mensah I.K., Zeng G., Mwakapesa D.S. (2022). The behavioral intention to adopt mobile health services: The moderating impact of mobile self-efficacy. Front. Public Health.

[47] Su J., Wang Y., Liu H., Zhang Z., Wang Z., Li Z. (2025). Investigating the factors influencing users’ adoption of artificial intelligence health assistants based on an extended UTAUT model. Sci. Rep..

[48] Brislin R.W. (1970). Back-translation for cross-cultural research. J. Cross-Cult. Psychol..

[49] Klotz A.C., Swider B.W., Kwon S.H. (2023). Back-translation practices in organizational research: Avoiding loss in translation. J. Appl. Psychol..

[50] Razin Y.S., Feigh K.M. (2024). Converging measures and an emergent model: A meta-analysis of human-machine trust questionnaires. ACM Trans. Hum.-Robot Interact..

[51] Balaskas S., Yfantidou I., Skandali D. (2025). Eyes on prevention: An eye-tracking analysis of visual attention patterns in breast cancer screening ads. J. Eye Mov. Res..

[52] Barone M., Bussoli C., Fattobene L., Ling A. (2025). Exploring attentional mechanisms underlying the gender homophily in equity crowdfunding decisions using web-based eye-tracking. Sci. Rep..

[53] Amin A., Shah B., Khattak A.M., Moreira F.J.L., Ali G., Rocha A., Anwar S. (2019). Cross-company customer churn prediction in telecommunication: A comparison of data transformation methods. Int. J. Inf. Manag..

[54] Hooper R. (2025). To adjust, or not to adjust, for multiple comparisons. J. Clin. Epidemiol..

[55] Li H., Li J., Hao X., Liu W. (2025). Behavioral and eye-tracking investigation of event segmentation following short video watching. Npj Sci. Learn..

[56] Ayers J.W., Poliak A., Dredze M., Leas E.C., Zhu Z., Kelley J.B., Faix D.J., Goodman A.M., Longhurst C.A., Hogarth M. (2023). Comparing physician and artificial intelligence chatbot responses to patient questions posted to a public social media forum. JAMA Intern. Med..

[57] Astobiza A.M., Alonso M., Ortega Lozano R. (2025). Trust and AI in healthcare: A systematic review. Monash Bioeth. Rev..

[58] Sagona M., Dai T., Macis M., Darden M. (2025). Trust in AI-assisted health systems and AI’s trust in humans. npj Health Syst..

[59] Romeo G., Conti D. (2025). Exploring automation bias in human–AI collaboration: A review and implications for explainable AI. AI Soc..

[60] Hu H., Li H., Wang B., Zhang M., Wu B., Wu X. (2024). Application of eye-tracking in nursing research: A scoping review. Nurs. Open.

[61] Specian Junior F.C., Litchfield D., Sandars J., Cecilio-Fernandes D. (2024). Use of eye tracking in medical education. Med. Teach..

[62] Onnasch L., Schweidler P., Schmidt H. (2023). The potential of robot eyes as predictive cues in HRI—An eye-tracking study. Front. Robot. AI.

[63] Li M., Guo F., Wang X., Chen J., Ham J. (2023). Effects of robot gaze and voice human-likeness on users’ subjective perception, visual attention, and cerebral activity in voice conversations. Comput. Hum. Behav..

[64] Park Y., Kim H., Kim H. (2024). Visualizing empathy in patient-practitioner interactions using eye-tracking technology: Proof-of-concept study. JMIR Form. Res..

[65] Janson A. (2023). How to leverage anthropomorphism for chatbot service interfaces: The interplay of communication style and personification. Comput. Hum. Behav..

[66] Lee J.R., Gutzwiller R.S. (2025). Do the eyes have it? A review of using eye tracking for automation trust measurement. Hum. Factors.

[67] Xu Z., Lee Y.C., Stasiak K., Warren J., Lottridge D. (2025). The digital therapeutic alliance with mental health chatbots: Diary study and thematic analysis. JMIR Ment. Health.

[68] Sanjeewa R., Iyer R., Apputhurai P., Wickramasinghe N., Meyer D. (2025). Perception of empathy in mental health care through voice-based conversational agent prototypes: Experimental study. JMIR Form. Res..

